# Effect of Intermittent Fasting (18/6) on Energy Expenditure, Nutritional Status, and Body Composition in Healthy Adults

**DOI:** 10.1155/2021/7809611

**Published:** 2021-12-18

**Authors:** Duygu Ağagündüz, Nilüfer Acar-Tek, Osman Bozkurt

**Affiliations:** ^1^Gazi University, Faculty of Health Sciences, Department of Nutrition and Dietetics, Ankara, Turkey; ^2^Erzurum Technical University, Faculty of Health Sciences, Department of Nutrition and Dietetics, Erzurum, Turkey

## Abstract

**Objective:**

This study was aimed at evaluating the effect of intermittent fasting of Ramadan on resting energy expenditure (REE), body composition, and nutritional status.

**Methods:**

The study was conducted on a total of 27 adults (16 females, 11 males) who were fasting (18 h) in the Ramadan month (May 6–June 3) of 2019. REE was measured using the indirect calorimeter. Dietary energy and nutrient intakes were evaluated by 3-day food records in baseline and post-Ramadan. Body composition and some metabolic parameters were analyzed simultaneously with REE measurements. All measurements were performed two times at baseline, and post-Ramadan.

**Results:**

Body weight (−2.9% vs. −1.4%), body mass index (BMI) (−3.1% vs. −2.1%), fat-free mass (−2.7% vs. −1.4%), and hydration status were decreased in both males and females after the Ramadan fasting (*p* < 0.05). REEs (kcal/d) of the participants were 1708.1 ± 262.50 kcal/d and 1596.5 ± 302.27 kcal/d at baseline and post-Ramadan, respectively (6.5%) (*p* < 0.05). This decrease in REE (kcal/d) in females was greater than that in males (−8.1% vs. −4.6%). However, no statistically significant difference was found in sleep duration (h), physical activity levels, dietary energy and nutrient intakes, and blood pressures (mm Hg) of both genders compared to baseline (*p* > 0.05).

**Conclusion:**

Intermittent circadian fasting may lead to a decreased energy expenditure and a change in fat-free mass in healthy individuals, and this effect is interpreted as gender-dependent.

## 1. Introduction

Intermittent fasting is an interventional strategy where participants are exposed to various fasting situations. Intermittent fasting is defined as the nutrition type, which causes a decrease in daily total energy intake (50–100%) due to food intake restriction in full or partial (e.g., 16–48 h) at certain times of the day, 1–3 times a week, or prolonged night time [1, 2]. Since ancient times, especially in the treatment of epilepsy, mainly in ketogenic diet applications, it has been used in various ways [3]. However, its popularity increased when its effect on the potential improvement of metabolic abnormalities was determined experimentally [4]. It is reported that it may have potentially positive effects on obesity, type 2 diabetes, cardiovascular disease, and some cancer types due to changes it causes in body weight and metabolic parameters. It can also be performed in association with circadian rhythm, intestinal microbiota, and lifestyle habits that can be modified such as diet, physical activity, and sleep [5]. It has also been reported that adaptation may be better in this type of diet than other dietary practices [6]. Nowadays, the ultimate goal of most intermittent fasting regimens is to improve body composition [7]. However, losing weight is not typically targeted in the intermittent fasting type of Ramadan.

Intermittent fasting has many types such as (i) complete alternate day fasting (full-fasting for some foods/drinks every other day), (ii) modified fasting regimen (restriction twice a week, 5 days ad libitum feeding), (iii) time-restricted feeding (<3 meals, prolonged day or night fasting), and (iv) religious fasting (Ramadan fasting and other religious fasts) [5, 8, 9]. For example, during the Ramadan month (the ninth month of the Islamic calendar), Muslims do not eat or drink anything during daylight hours, eating one light meal (the “sahur,” “suhoor,” or “sehri”) just before dawn and another (the “iftar”) after sunset 29–30 days [10].

Fasting means avoiding food intake for a certain time depending on religious and/or spiritual traditions. Although it changes with the religions/traditions, depending on the season and geographic location, the restriction period for food intake varies between 12 h and 4 weeks [11]. It is reported that fasting may cause a 0–40% decrease in daily energy intake. However, the frequency of meals usually remains the same in this diet [12]. On the other hand, changes might occur in fluid intake and hydration [13]. For these reasons, changes in body weight may occur during the Ramadan period. A meta-analysis study reported that the average body weight loss during the Ramadan period could be 1.24 kg and 0.76 kg body weight gain within 2 weeks following Ramadan [12]. In a recent meta-analysis, Ramadan intermittent fasting is effective in decreasing body weight (−0.353; 95% CI [−0.651, −0.054]) and relative fat mass (−0.533; 95% CI [−1.025, −0.04]) [7].

The mechanism by which intermittent fasting or Ramadan fasting caused these changes in body weight is still unclear. There are very few studies in the literature on the effect of intermittent food intake on energy expenditure [1, 14]. Animal studies have demonstrated that there may be an improvement in muscle coordination, increased activity, and increase in energy expenditure in the wake of time-limited isocaloric nutrition practices [14]. In a study, the effect of intermittent/restricted diet for 8 weeks (16/8) on basal metabolic rate (BMR) in males who perform resistant exercise was evaluated and it was reported that there was no change in thyroid-stimulating hormone (TSH) level, but the level of triiodothyronine (T3) decreased in resting energy expenditure (REE) [1]. These mentioned food intakes and body composition changes are desired in some cases; however, they may also cause undesirable results via these underlying mechanisms. So, more research is needed on the effect of intermittent fasting and/or Ramadan fasting on energy expenditure and its mechanism of effect in humans.

We hypothesized that Ramadan fasting might affect body composition and REE. This is the first study to evaluate the effect of intermittent fasting status on REE, body composition, and nutritional status in the 18-hour period of intermittent fasting (18/6) during the Ramadan month in the Turkish population.

## 2. Materials and Methods

### 2.1. Study Group

The study was conducted on 27 Turkish adult participants, 16 females and 11 males, who were fasting in Ankara, Turkey. Participants were selected among participants who were fasting and nonsmokers, did not consume alcohol and did not perform high levels of physical activity or were not athletes. Those with endocrine and metabolic disorders diagnosed by the physician, those with respiratory diseases such as asthma, those who had influenza, the flu, cold when resting energy expenditure (REE) measurement protocol was performed, and people on regular medications and/or nutritional supplements and not fasting were not included in the study. Besides, those who were pregnant and breastfeeding, and individuals with concerns in adapting to the research were not included in the study. In the selection of females included in the study, it was cared not to coincide with menstrual cycles in this study period because females must not have fasting in the menstruation period in Ramadan month because of religious beliefs.

**Figure 1:**
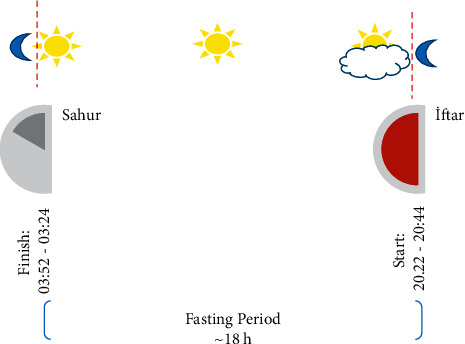
Schema of Ramadan fasting.

The research was carried out in the Nutrition Anthropometry Laboratory of Gazi University Nutrition and Dietetics Department during the Ramadan month (from May 6, 2019 to June 3, 2019). This fasting period is shown in Figure 1.

### 2.2. Ethical Considerations

Ethics committee approval (77082166-604.01.02-08/01/2019) was obtained from Gazi University Clinical Research Ethics Committee to carry out this study. Furthermore, participants have been explained the purpose and method of the study, and participants who accepted to participate in the study were signed a voluntary consent form by following the Helsinki Declaration (World Medical Association).

### 2.3. Determination of the General Characteristics

A questionnaire was used to question the participants' sociodemographic characteristics and health histories with a face-to-face interview technique. Besides, through this questionnaire, physical activity status and sleep habits changes (sleep duration was asked) were followed in the 18-hour period of intermittent fasting during the Ramadan month (18/6).

### 2.4. Evaluation of Nutritional Status

To evaluate the participants' daily dietary energy and nutrient intake, 24-hour dietary recall (for 3 sequential days) was taken before the Ramadan/intermittent fasting (baseline) and post-Ramadan/intermittent fasting, one of which would coincide with the weekend. In the assessment of daily intake of dietary energy and nutrients, the “Computer-based Nutrition Program, Nutrition Information System- BEBIS (8.1)” program adapted for Turkey was used [15].

### 2.5. Measurements

#### 2.5.1. Resting Energy Expenditure (REE)

REE was measured using the Cosmed Fitmate indirect calorimeter (Cosmed, Rome, Italy). REE measurements were performed two times before intermittent fasting (baseline) and post-Ramadan period. The measurements were taken before and after the Ramadan fasting period between 08.00 and 10.00 am. The participants, who were to be measured for REE, were requested not to do the heavy exercise one day before and maintain their diet as usual. The participants were allowed to rest in the sitting position for 15 mins before the test. To identify the average oxygen consumption of participants (VO_2_), the average ventilation (Ve), the average respiratory frequency (Rf), and the average oxygen concentration in the exhaled air (FeO_2_), the measurement was conducted with a mask covering the nose entirely in a supine position for 15 mins in a silent and thermoneutral (22–24°C) environment, with complete resting and supine position [16].

#### 2.5.2. Body Composition Analysis

Body composition analysis of the participants was conducted before morning REE measurement was taken (08.00–10.00 am) through InBody720 Body Composition Analyzer (body weight (kg), body fat mass (kg), body fat percentage (%), fat-free mass (kg), total body water (L), intracellular water (L), extracellular water (L)). Anthropometric measurements such as body weight (kg), waist circumference (cm), and hip circumference (cm) were obtained, and waist-to-hip ratios were calculated. Anthropometric measurements and body composition analysis were performed two times before Ramadan/intermittent fasting (baseline) and the post-Ramadan.

#### 2.5.3. Evaluation of Hydration Status

In the analyses of the participants with InBody720 Body Composition Analyzer, the effect of Ramadan/intermittent fasting was evaluated by determining the changes in total body water (L), intracellular water (L), and extracellular water (L) considered to be hydration markers in terms of the study.

#### 2.5.4. Measurement of Other Metabolic Parameters

Systolic blood pressure and diastolic blood pressure measurements (mm Hg) as metabolic/vital parameters were performed simultaneously while measuring REE.

### 2.6. Evaluation of Physical Activity Levels

To evaluate the physical activity status of the participants, 24 h of physical activity record was taken before the Ramadan/intermittent fasting (baseline) period and post-Ramadan. The form gave information regarding activity type, level, and duration. Mean physical activity levels (PAL) were derived from activity duration *x* physical activity ratio (PAR) provided by the “Report of a Joint FAO/WHO/UNU Expert Consultation” divided by 24 h. PAL was classified as sedentary and light activity 1.40–1.69, active or moderately active 1.70–1.99, and energetic or heavily active 2.00–2.40 [17]. Total energy expenditure (TEE) of participants is calculated by multiplying REE and PAL.

### 2.7. Statistical Analysis

The statistical evaluation of the data was performed by using the Statistical Package for the Social Sciences (SPSS, version: 22.0) statistical package program. Percentage and arithmetic mean ± standard deviation (*x̅* ± SD) values are given for the measured variables.

Visual and analytical methods (Kolmogorov–Smirnov/Shapiro–Wilk tests) were used to evaluate the convenience of data to normal distribution. Wilcoxon matched-pair signed-rank test was applied to compare the body composition, energy expenditure, and some metabolic parameters, and also daily dietary energy and macronutrient intakes of participants before the Ramadan/intermittent fasting (baseline) and post-Ramadan period. Besides, the changes in body composition, energy expenditure, and metabolic parameters of the participants because of the Ramadan/intermittent period were expressed as a percentage (%) of difference. A multiple linear regression model was used to identify independent predictors of post-Ramadan REE. Statistical significance was defined as *p* < 0.05.

## 3. Results

### 3.1. General Characteristics and Dietary Intakes

A total of 27 participants, 11 males, and 16 females, aged 27.6 ± 1.69 y, completed the study and had fully paired (baseline and 28th day of Ramadan) acquired all data. The dietary energy and nutrient intakes of the participants at baseline and post-Ramadan are shown in Table 1. Accordingly, the dietary energy intake of overall participants was determined as 1549.4 ± 429.05 kcal/d and 1530.1 ± 473.72 kcal/d at baseline and post-Ramadan, respectively (*p* > 0.05). Dietary protein intake was 66.5 ± 18.19 g/d at baseline, whereas it was 58.0 ± 18.74 g/day post-Ramadan (*p* > 0.05). On the contrary of this result, dietary protein intake of females was statistically significant decreased (66.8 ± 19.40 vs. 51.9 ± 16.52 g) at the end of the Ramadan compared to baseline (*p* < 0.05) Fat intake was found to be 69.9 ± 23.98 g/d and 70.4 ± 27.04 g/d, whereas carbohydrate intake was 161.5 ± 50.83 g/d and 162.4 ± 52.93 g/d at baseline and post-Ramadan, respectively (*p* > 0.05) (Table 1). No statistically significant difference was found in both genders' dietary fat and carbohydrate intakes after the Ramadan (*p* > 0.05). Furthermore, there was no statistically significant difference between the number of participants' meals at baseline and post-Ramadan (data not shown in the table).

### 3.2. Body Composition Changes

Body compositions of the participants at baseline and post-Ramadan period are shown in Table 2. Body weights of the overall participants were 70.5 ± 14.46 kg and 69.0 ± 13.99 kg at baseline and post-Ramadan, respectively (−2.1%) (*p* < 0.05). Overall BMI (kg/m^2^) value was 24.1 ± 3.67 kg/m^2^ at baseline, whereas it was 23.5 ± 3.68 kg/m^2^ post-Ramadan (−2.4% reduction). It was found that body weight (−2.9% vs. −1.4%) and BMI (−3.1% vs. −2.1%) were significantly lower in both males and females at the post-Ramadan period compared to baseline (*p* < 0.05).

Body fat mass (kg) was determined as 19.8 ± 9.36 kg at baseline and 19.5 ± 9.60 kg post-Ramadan (*p* > 0.05). Body fat percentage (%) was 27.6 ± 9.54% and 27.7 ± 9.95% at baseline and post-Ramadan, respectively (*p* > 0.05). Body fat mass (kg) and body fat percentage (%) of both males and females at baseline and post-Ramadan were not statistically different (*p* > 0.05) (Table 2). However, the difference between fat-free mass (50.7 ± 11.13 vs. 49.7 ± 10.77 kg, [−1.9%]) at baseline and post-Ramadan period was statistically significant (*p* < 0.05). It was determined that fat-free mass was significantly lower in both males and females after the Ramadan period (−2.7% vs. −1.4%) (*p* < 0.05) (Table 2).

When hydration parameters of the participants at baseline and post-Ramadan were evaluated (Table 2), total body water (l) was found to be 36.7 ± 8.70 l and 35.5 ± 8.19 l for all participants at baseline and post-Ramadan, respectively (−3.2%). The difference between extracellular fluid amounts (23.2 ± 5.31 22.6 ± 5.10 l, [−2.6%]) and intracellular fluid amounts (13.9 ± 2.84 l and 13.7 ± 2.79 l, [−1.4%]) in overall participants at baseline and post-Ramadan was statistically significant (*p* < 0.05). Besides, the changes in total body water (L) intracellular and extracellular fluid amounts in males after the Ramadan period was more remarkable than those in females (*p* < 0.05).

### 3.3. Energy Expenditure and Metabolic Parameters

Changes in the sleep duration, energy expenditure, and some metabolic parameters over time after Ramadan fasting according to genders are evaluated in Table 3.

Total sleep durations of participants were found as 7.2 ± 0.85 h and 7.4 ± 1.29 at baseline and post-Ramadan, respectively (*p* > 0.05). Also, the total sleep duration (h) of both genders because of Ramadan was not statistically different compared to the baseline period (*p* > 0.05) (Table 3).

REE (kcal/d) values of the participants were 1708.1 ± 262.50 kcal/d and 1596.5 ± 302.27 kcal/d at baseline and post-Ramadan, respectively (−6.5%) (*p* < 0.05). Although REEs of both males and females at post-Ramadan periods were statistically significantly lower compared to baseline (*p* < 0.05), the decrease in REE (kcal/d) in females after the Ramadan period was greater than males (−8.1% vs. −4.6%). Likewise, the baseline VO_2_ value (248.2 ± 37.02 mL/min) became 230.9 ± 43.26 mL/min at post-Ramadan period (−6.9%) (*p* < 0.05). In females, VO_2_ (mL/min) values were 228.6 ± 25.59 mL/min and 206.2 ± 24.90 mL/min at baseline and post-Ramadan, respectively (−9.8%) (*p* < 0.05). However, the changes in VO_2_ values over time were not statistically significant in males (*p* > 0.05). No statistically significant difference was also found between Ve (L/min), Rf (L/min), and FeO_2_ (%) values of the participants at baseline and post-Ramadan (*p* > 0.05) (Table 3).

When physical activity levels of the participants were evaluated (Table 3), there was no statistically significant difference between energy expenditure for physical activity (kcal/d) and PAL values of the participants at baseline and on post-Ramadan (*p* > 0.05) (Table 3). However, the evaluation of the TEE (kcal/d) values of the participants by following the Ramadan fasting showed that baseline TEE (kcal/d) values (2193.1 ± 404.12 kcal/d) became 1920.6 ± 318.41 kcal at post-Ramadan (−12.4%) (*p* < 0.05). Also, the decrease in TEE (kcal/d) in males was greater than in females (−15.2% vs. −9.4%), and there was a statistically significant difference between TEE values (kcal/d) of males at baseline and post-Ramadan (*p* < 0.05).

No statistically significant difference was found between the blood pressure parameters of all participants before and post-Ramadan (*p* < 0.05) (Table 3).

A multiple linear regression model related to post-Ramadan REE is presented in Table 4 (Table 4). Among all dependent variables, baseline REE and energy related to physical activity could predict post-REE and that energy related to physical activity had a great impact (Table 4).

## 4. Discussion

This study was planned and conducted to evaluate the effect of intermittent fasting status on REE, body composition, and nutritional status in the 18-hour period of intermittent fasting (18/6) after Ramadan. To the best of our knowledge, this was the first time examining these effects, especially energy expenditure in healthy adults due to intermittent fasting of Ramadan in Turkey.

The first question in this study sought to determine changes in nutritional status by the following Ramadan fasting period. Until recently, there has been no clear evidence of the effects of intermittent fasting on individuals' dietary energy and nutrient intakes [18–23]. The current study found that some changes were observed in the energy and nutrient intakes of the participants at the end of the Ramadan period. However, the observed differences except for protein intakes between baseline and post-Ramadan in this study were insignificant (Table 1). Interestingly, only females statistically significantly had less dietary protein intake after the Ramadan period (Table 1). This interpretation differed from that of a study conducted on 240 adults, which suggested that dietary protein intake was determined to decrease only in male participants [18].

Furthermore, a study reported no difference in energy and nutrient intakes of young women during Ramadan fasting despite the decrease in the number of meals [19]. Another study in individuals with type 2 diabetes noted that total energy, energy from carbohydrates, and protein intake after Ramadan fasting were similar to those before Ramadan, while energy from fat intake of individuals increased [20]. Also, it showed that total energy intake during Ramadan fasting did not differ from baseline while high fat and protein increased [24]. By contrast, it was determined that there was an increase in daily energy intake during Ramadan, but fat, fatty acids, and cholesterol intake decreased parallelly with the decrease in the number of meals in healthy young individuals [21].

Besides the changes in energy and nutrient intakes, a study found that Ramadan fasting caused changes in the food pattern and dietary diversity in adolescents [22]. Another study suggests that adolescents' motivation to fast during Ramadan was due to spiritual decisions rather than weight control or other factors [25]. In the present study, it is thought that the fact that the number of meals does not change may be the main reason for the lack of difference in dietary energy and nutrient intakes. Because losing weight is not generally targeted in intermittent fasting of Ramadan, and there is no restriction on the total number of meals. Also, this discrepancy could be attributed to differences in age groups and health status.

One of the most important findings of this study was the decrease in body weight (−2.9% vs. −1.4%), BMI (−3.1% vs. −2.1%), and fat-free mass (−2.7% vs. −1.4%) in both males and females after Ramadan fasting (Table 2). The impact of intermittent fasting programs on body composition and disease-related clinical/health parameters is not clear. Prior studies showed these effects might vary according to the intermittent fasting type (26–28). A study showed that implementing an alternate fasting program in normal-weight, overweight, and obese participants for 3 to 12 weeks resulted in a decrease in body weight (3–7%) and body fat (3–5.5 kg) and improvements in blood lipid profile. In addition, whole-day fasting practice was also reported to cause reductions in body weight (3–9%) and body fat over for 12–24 weeks, resulting in a reduction in total cholesterol (5–20%) and triglyceride (17–50%) levels [26]. Another study evaluating the effect of Ramadan fasting in healthy men, significant reductions in body weight, BMI, skeletal muscle mass, and lean body mass was found similar to the current study [27]. Moreover, no significant effects were observed on mood, appetite, and quality of life parameters [27]. On the contrary, a 14-hour fasting type for 28 days caused minor changes in BMI without greatly affecting the body composition, glucose metabolism, and cognitive function (−0.2 kg/m^2^) in another study evaluating the effect of intermittent fasting in the Ramadan model on body composition in healthy lean men [28]. A recent meta-analysis showed significant decreases in body weight with Ramadan intermittent fasting [7]. In a systematic review and meta-analysis study evaluating the effects of Ramadan fasting in healthy participants, body weight loss was correlated with BMI. It was also reported significant reductions in body fat percentage (1.46%) in overweight or obese participants but not in normal-weight participants [29]. It was even noted that the participants returned to pre-Ramadan body weight and composition in 2–5 weeks after the end of Ramadan [29]. Likewise, a study showed that adolescents regained their lost weight (∼1.5 kg) 1 month after Ramadan [23]. Taken together, there is also a risk of regaining weight after Ramadan type of fasting [23, 29]. It is considered that other intermittent fasting programs should not be generalized to Ramadan fasting program.

Another important finding of the present study was that body composition changes in male participants due to Ramadan fasting tended to be more extensive than females (Table 2). The literature supported that changes in body composition caused by Ramadan fasting varied depending on gender and age [7, 18, 30]. Similar to this, in a meta-analysis evaluating the effect of Ramadan fasting on body weight in healthy participants, it was found that Ramadan fasting changed body weight especially in males [30]. Another study on adults reported that Ramadan fasting provoked loss in body weight and lean body mass, but the change in BMI and body composition due to Ramadan fasting was the highest in males under 35 years of age [18].

The most surprising result in this study was that the total sleep duration of both genders because of Ramadan was not statistically different compared to the baseline period (*p* > 0.05) (Table 3). In fact, Ramadan is a fasting practice in which changes in sleep and activity occur, often accompanied by changes in the release of certain hormones associated with the circadian rhythm [31]. Changes in activity status and sleeping habits may occur in Ramadan [31]. The effects of experimental fasting practices on cognitive function, sleep, and wakefulness status have been shown in previous studies [27, 32, 33]. However, it is suggested that experimental fasting practices should not be generalized to Ramadan fasting practice in the literature [33]. The rapid eye movement (REM) sleep proportion could be decreased after fasting practices and therefore causes significant changes in sleep architecture. However, well-designed studies have also shown that fasting practice does not affect circadian rhythm [33]. Moreover, when another study evaluated the effects of Ramadan fasting on insomnia parameters by dividing the Ramadan into three-phase (before, middle, and last periods), it was determined that insomnia during the day improved, especially from the middle to the last days of Ramadan [27]. So, no difference was thought to be observed in total sleep durations between before and post-Ramadan because the sleep duration at the baseline and end of Ramadan was only evaluated in this study.

In the present study, PALs and energy expenditure values for physical activity in both male and female participants were not found to be statistically different before and post-Ramadan (*p* > 0.05) (Table 3). In the same way, activity status during Ramadan fasting (PAL: 1.54 vs. 1.51) did not change compared to the period before Ramadan in young women [20]. However, it is often reported that Ramadan fasting increases sleepiness during the day and may reduce physical activity [34]. In addition to activity status, the effect of Ramadan fasting on hydration status has been investigated in the literature [35, 36]. In a systematic review, BMI, body fat, and body fat percentage during Ramadan have been reported to be lower compared to the baseline period. Still, lean mass and total body water did not change during Ramadan in subjects [35]. However, literature mainly indicated that Ramadan fasting could induce dehydration, marked by an increase in blood measures and hydration markers, but no detrimental effects on health have yet been directly attributed to negative water balance, at the levels that may occur during Ramadan [36]. Likewise, in the current study, it was found that total body water decreased in both male and female participants after Ramadan fasting (*p* < 0.05) and the changes in total body water, intracellular and extracellular fluid amounts (L), in males after Ramadan period was greater than females (*p* < 0.05) (Table 2). It is thought that this may be caused especially by the loss of fat-free mass in participants and this fluid loss may be a risk for weight regain after the Ramadan period.

One of the most striking findings of this study was that REE (kcal/d) levels were significantly decreased in females, whereas TEE (kcal/d) levels were significantly decreased in males after Ramadan fasting (*p* < 0.05) (Table 3). Although there is some evidence reporting the positive effects of intermittent fasting on body weight and health markers, as mentioned before, the effects of intermittent fasting on energy balance and energy expenditure have not yet been clarified [31, 37]. In a randomized controlled trial on adult participants (30–36 lean and 30–36 overweight/obese participants), (i) daily calorie restriction (reducing habitual daily energy intake by 25%), (ii) intermittent fasting with calorie restriction (alternating between 24-hour periods of fasting and feeding to 150% of habitual daily energy intake), and (iii) intermittent fasting without calorie restriction (alternating between 24-hour periods of fasting and feeding to 200% of habitual daily energy intake) were performed [38]. As a result of the study, it was found that all practices of intermittent fasting affect postprandial metabolism and energy balance components in both lean and overweight/obese participants [38]. By contrast, there is some evidence that REE and TEE did not change by Ramadan fasting [31, 37]. Supporting this, a study determined that Ramadan fasting decreased activity level and sleep duration but no change in REE and TEE levels in nonobese healthy adults. Besides, it was reported that weight losses mainly occurred due to changes in dietary intakes [37]. On the contrary, in the present study, as an exciting finding, the most important determiners of post-Ramadan REE (kcal/d) were found as baseline REE (kcal/d) levels and physical activity markers in the multiple linear regression model (Table 4). So, this study has shed light on how Ramadan fasting changes energy expenditure regardless of many factors such as dietary intake, body composition, and activity status.

Ramadan fasting is the state of intermittent glycogen depletion and repletion. However, the precise mechanism of the effects of Ramadan fasting on substrate oxidation as well as on REE (kcal/d) and TEE (kcal/d) remains not to be elucidated. While carbohydrates are used as the primary fuel source in the early morning hours, lipids become more important in the afternoon and especially when it is about to be iftar time [31]. The effect of Ramadan fasting on substrate oxidation was not evaluated in the current study. Therefore, further research in this field would be a great help for understanding changes in energy expenditures because of intermittent fasting practice.

Also, it is stated that Ramadan intermittent fasting reduces cardiac stress among hypertensive patients [39]. However, in our study, blood pressure was no different between before and post-Ramadan. Since this study was conducted in healthy individuals, it may not have detected a difference.

## 5. Conclusions

In conclusion, it can be concluded that intermittent fasting of Ramadan can lead to undesirable decrease in REE (kcal/d)-TEE (kcal/d), and also alter BMI (kg/m^2^) and body composition especially in fat-free mass, and hydration status, even if the participants are healthy. These effects can be varied in different genders. No significant effects on dietary energy and nutrients were determined after Ramadan fasting. So, the empirical findings in this study provided a new understanding of the effects of intermittent fasting of Ramadan on energy expenditure, body composition, and nutritional status. However, the generalization of the results of study may be limited in people with disease history and to other cultures in which the fasting practices of Ramadan might be different.

In future studies, Ramadan fasting with physical activity in a larger sample group is of interest in changing body composition and energy expenditure. Besides, it may be considered to include the population with having different health problems, but it is obvious that long-term fasting is not easy, especially in people with certain metabolic diseases. Also, Ramadan fasting' effects on body composition and TEE need to be evaluated long term.

## Figures and Tables

**Table 1:** Dietary energy and nutrient intakes of the participants at baseline and post-Ramadan.

Energy and nutrient intakes	Males (*n*: 11)			Females (*n*: 16)			Overall (*n*: 27)		
	Baseline	Post-Ramadan	Sig.	Baseline	Post-Ramadan	Sig.	Baseline	Post-Ramadan	Sig.
	*x̅* ± SD	x̅ ± SD		*x̅* ± SD	*x̅* ± SD		*x̅* ± SD	*x̅* ± SD	
Energy (kcal/d)	1618.9 ± 349.49	1821.2 ± 448.02	NS	1503.0 ± 480.93	1283.8 ± 345.58	NS	1549.4 ± 429.05	1530.1 ± 473.72	NS
Protein (g/d)	66.1 ± 17.24	65.3 ± 19.31	NS	66.8 ± 19.40	51.9 ± 16.52	^*∗*^	66.5 ± 18.19	58.0 ± 18.74	NS
Fat (g/d)	72.0 ± 11.10	87.0 ± 26.05	NS	68.6 ± 30.02	56.4 ± 19.24	NS	69.9 ± 23.98	70.4 ± 27.04	NS
Carbohydrate (g)	172.8 ± 51.02	190.6 ± 50.60	NS	154.0 ± 51.04	138.5 ± 43.41	NS	161.5 ± 50.83	162.4 ± 52.93	NS

NS: not significant, ^*∗*^*p* < 0.05.

**Table 2:** Body composition of the participants at baseline and post-Ramadan.

Variables	Males (*n*: 11)				Females (*n*: 16)				Overall (*n*: 27)			
	Baseline	Post-Ramadan	Difference (%)	Sig.	Baseline	Post-Ramadan	Difference (%)	Sig.	Baseline	Post-Ramadan	Difference (%)	Sig.
	*x̅* ± SD	*x̅* ± SD			*x̅* ± SD	*x̅* ± SD			*x̅* ± SD	*x̅* ± SD		
*Body composition parameters*												
Body weight (kg)	81.5 ± 13.50	79.1 ± 13.69	−2.9	^*∗*^	63.0 ± 9.58	62.1 ± 9.39	−1.4	^*∗*^	70.5 ± 14.46	69.0 ± 13.99	−2.1	^*∗*^
BMI (kg/m^2^)	25.5 ± 3.75	24.7 ± 3.87	−3.1	^*∗*^	23.1 ± 3.39	22.6 ± 3.41	−2.1	^*∗*^	24.1 ± 3.67	23.5 ± 3.68	−2.4	^*∗*^
Body fat mass (kg)	18.7 ± 11.18	18.1 ± 11.06	−3.2	NS	20.5 ± 8.19	20.4 ± 8.71	−0.4	NS	19.8 ± 9.36	19.5 ± 9.60	−1.5	NS
Body fat percentage (%)	22.0 ± 8.54	22.0 ± 8.33	—	NS	31.5 ± 8.40	31.6 ± 9.22	+0.3	NS	27.6 ± 9.54	27.7 ± 9.95	+0.3	NS
Fat-free mass (kg)	62.6 ± 6.57	60.9 ± 6.79	−2.7	^*∗*^	42.6 ± 3.52	42.0 ± 3.80	−1.4	^*∗*^	50.7 ± 11.13	49.7 ± 10.77	−1.9	^*∗*^
Waist-to-hip ratio	0.9 ± 0.06	0.8 ± 0.07	−11.1	^*∗*^	0.8 ± 0.06	0.8 ± 0.06	—	^*∗*^	0.8 ± 0.06	0.8 ± 0.06	—	^*∗*^

*Hydration parameters*												
Total body water (L)	45.2 ± 4.79	42.5 ± 8.52	−6.6	^*∗*^	30.4 ± 3.63	30.7 ± 2.78	+0.9	NS	36.7 ± 8.70	35.5 ± 8.19	−3.2	^*∗*^
Intracellular water (L)	28.9 ± 3.14	27.9 ± 3.23	−3.5	^*∗*^	19.3 ± 1.60	18.9 ± 1.71	−2.0	^*∗*^	23.2 ± 5.31	22.6 ± 5.10	−2.6	^*∗*^
Extracellular water (L)	16.8 ± 1.70	16.6 ± 1.72	−1.1	^*∗*^	11.8 ± 1.03	11.7 ± 1.09	−0.8	NS	13.9 ± 2.84	13.7 ± 2.79	−1.4	^*∗*^

BMI: body mass index; NS: not significant, ^*∗*^*p* < 0.05.

**Table 3:** Energy expenditure and some metabolic parameters of the participants at baseline and post-Ramadan.

Variables	Males (n:11)				Females (n:16)				Overall (n:27)			
	Baseline	Post-Ramadan	Difference (%)	Sig.	Baseline	Post-Ramadan	Difference (%)	Sig.	Baseline	Post-Ramadan	Difference (%)	Sig.
	x̅±SD	x̅±SD			x̅±SD	x̅±SD			x̅±SD	x̅±SD		
Sleep duration (h)	6.8 ± 0.77	6.8 ± 1.05	—	NS	7.5 ± 0.81	7.7 ± 1.34	+2.6	NS	7.2 ± 0.85	7.4 ± 1.29	+2.7	NS

*Energy expenditure parameters*												
REE (kcal/d)	1927.7 ± 228.51	1838.8 ± 289.10	−4.6	NS	1557.2 ± 158.05	1430.0 ± 173.27	−8.1	^*∗*^	1708.1 ± 262.50	1596.5 ± 302.27	−6.5	^*∗*^
VO_2_ (mL/min)	276.8 ± 32.67	266.3 ± 40.05	−3.8	NS	228.6 ± 25.59	206.2 ± 24.90	−9.8	^*∗*^	248.2 ± 37.02	230.9 ± 43.26	−6.9	^*∗*^
Ve (L/min)	8.8 ± 1.24	8.4 ± 1.50	−4.5	NS	7.8 ± 1.77	7.4 ± 1.40	−5.1	NS	8.2 ± 1.63	7.8 ± 1.49	−4.8	NS
Rf (L/min)	15.7 ± 4.51	16.0 ± 4.04	+1.9	NS	16.5 ± 3.13	16.6 ± 4.30	+0.6	NS	16.2 ± 3.69	16.3 ± 4.12	+0.6	NS
FeO_2_ (%)	16.9 ± 0.32	16.8 ± 0.29	−0.6	NS	17.1 ± 0.48	17.3 ± 0.46	+1.1	NS	17.0 ± 0.44	17.1 ± 0.45	+0.5	NS
Energy expenditure for physical activity (kcal/d)	555.7 ± 100.98	333.3 ± 392.52	−40.0	NS	438.3 ± 273.55	377.6 ± 70.15	−13.8	NS	484.9 ± 224.43	360.5 ± 242.69	−25.6	NS
PAL value	1.2 ± 0.03	1.1 ± 0.21	−8.3	NS	1.2 ± 0.16	1.2 ± 0.04	—	NS	1.2 ± 0.12	1.2 ± 0.13	—	NS
TEE (kcal/d)	2480.4 ± 303.87	2101.5 ± 379.41	−15.2	^*∗*^	1995.6 ± 345.51	1807.6 ± 217.15	−9.4	NS	2193.1 ± 404.12	1920.6 ± 318.41	−12.4	^*∗*^

*Metabolic parameters*												
SBP (mm Hg)	104.9 ± 7.53	100.0 ± 19.37	−3.8	NS	73.9 ± 10.67	98.8 ± 9.67	+33.6	NS	102.7 ± 11.98	99.3 ± 14.2	−3.3	NS
DBP (mm Hg)	68.0 ± 8.11	77.7 ± 14.50	+12.9	NS	73.9 ± 10.67	71.0 ± 8.67	−3.9	NS	71.5 ± 9.99	73.8 ± 11.72	+3.2	NS

VO_2:_ average oxygen consumption; Ve: average ventilation; Rf: average respiratory frequency; FeO_2:_ average oxygen concentration in exhaled air; PAL: physical activity level; TEE: total energy expenditure; SBP: systolic blood pressure; DBP: diastolic blood pressure. NS: not significant, ^*∗*^*p* < 0.05.

**Table 4:** Multiple linear regression model of post-Ramadan resting energy expenditure.

Effect of characteristics	Post-Ramadan REE (kcal/d)			
	*β*	S.E	*t*	Sig.
Constant	9551.281	2471.695	3.864	^*∗*^
Baseline REE (kcal/d)	−0.883	0.232	−3.805	^*∗*^
Age (y)	−19.243	15.517	−1.240	NS
Gender	33.102	116.902	0.283	NS
Sleep duration (h/d)	−1.792	38.188	−0.047	NS
BMI (kg/m^2^)	2.631	23.118	0.114	NS
Body fat mass (kg)	8.269	10.371	0.797	NS
Fat-free mass (kg)	63.507	82.147	0.746	NS
Energy (kcal/d)	−2.054	1.410	−0.506	NS
Protein (g/d)	6.075	6.209	−1.457	NS
Fat (g/d)	16.514	11.845	0.979	NS
Carbohydrate (g)	9.165	5.817	1.575	NS
Energy expenditure for physical activity (kcal/d)	4.352	0.921	4.725	^*∗*^

REE: resting energy expenditure; BMI: body mass index; SE: standard error; NS: not significant, ^*∗*^*p* < 0.05. Dependent variable = post-Ramadan REE (kcal/d). Adjusted *R*^2^ = 0.898.

## Data Availability

The data that support the findings in this study are available from the corresponding author upon reasonable request.
